# Electrohydrodynamic convection instabilities observed in suspensions of cellulose nanocrystals

**DOI:** 10.1007/s10570-023-05391-6

**Published:** 2023-07-26

**Authors:** Bruno Frka-Petesic, Bruno Jean, Laurent Heux

**Affiliations:** 1 Yusuf Hamied Department of Chemistry, University of Cambridge, Lensfield Road, Cambridge, CB2 1EW UK; 2 International Institute for Sustainability with Knotted Chiral Meta Matter (WPI-SKCM²), 1-3-1 Kagamiyama, Hiroshima University, Higashi-Hiroshima, 739-8526 Japan; 3 Univ. Grenoble Alpes, CNRS, CERMAV, 38000 Grenoble, France

**Keywords:** Cellulose nanocrystal, Colloidal liquid crystal, Cholesteric, Electroconvection

## Abstract

Cellulose nanocrystals are slender, negatively charged nanoparticles that spontaneously form a cholesteric liquid crystal in aqueous suspension above a critical concentration. When they are suspended in apolar solvents such as toluene using surfactants, the application of an AC electric field leads to the reorientation and then distortion of the cholesteric order until the cholesteric structure completely unwinds into a nematic-like order, typically above 0.4–0.6 kV/cm at 1kHz. In this work, we show that at much higher electric fields ($\ge$ 4.6 kV/cm at 1 kHz) the sample develops a periodic pattern that varies with the field amplitude. We ascribed this pattern to electrohydrodynamic convection instabilities. These instabilities usually present complex regimes varying with the field, the voltage, the frequency and the geometry. However, the typical geometry where these instabilities were most documented across the literature differs from the geometry used in this work. This work concludes with possible future experimental investigations to clarify the exact regime of instability responsible for these observations.

**Supplementary Information:**

The online version contains supplementary material available at 10.1007/s10570-023-05391-6.

## Introduction

Cellulose nanocrystals (CNCs), as extracted from cotton by sulfuric acid hydrolysis, form colloidally stable aqueous suspensions capable of forming spontaneously a cholesteric liquid crystalline phase above a threshold concentration (Revol et al. 1992). The extension of their colloidal stability range to apolar solvents, achieved using suitable surface functionalization or surfactants, allows for the exploration of the formation of cholesteric phases where electrostatic interactions do not play a major role (Heux et al. 2000; Elazzouzi-Hafraoui et al. 2009). Moreover, this allows for the investigation of the individual and collective behavior of CNCs in suspensions in presence of strong electric fields (> 0.1 kV/cm) on centimeter-scale samples, without triggering the electrochemical complications due to water electrolysis. Previous works established that in dilute suspensions, CNCs align parallel to the electric field (Bordel et al. 2006), and that their alignment dynamics is dominated by a permanent dipole contribution when the electric field is suddenly reversed (Frka-Petesic et al. 2014). In cholesteric suspensions, the application of an electric field tends to first reorient the cholesteric domains so that their helical axes point away from the field direction, and then the helical order distorts to progressively align the CNCs along the field direction, leading to a pitch increase. Above a critical field value typically about 0.4–0.6 kV/cm, the cholesteric completely unwinds and exhibits a uniform monodomain nematic order (Frka-Petesic et al. 2017), following the behavior initially predicted theoretically and observed experimentally for molecular cholesteric systems and under magnetic fields when the molecules tend to align parallel to the magnetic field (De Gennes 1968; Rondelez and Hulin 1972), and later also experimentally observed in colloidal systems (Dogic and Fraden 2000). However, the size of the CNCs is orders of magnitude bigger than their counterparts in molecular liquid crystals, which leads to much larger electric dipoles and slower relaxation times, contributing to making apolar CNC dispersions an original system when exposed to strong AC electric fields (Frka-Petesic et al. 2014).

The effects of electric fields on the alignment of a nematic or cholesteric liquid crystal has been widely explored, especially in the geometry where the sample is confined in a narrow gap between two transparent electrodes (Blinov 1998). In this case, the field is applied in the viewing direction, and shows a variety of phenomena, depending on the anchoring conditions at the electrodes (either planar or perpendicular), the dielectric and conductive couplings of the field with the nematic orientation (either parallel or perpendicular), the confining gap and any symmetry-breaking element such as, e.g., an additional in-plane magnetic field (again with either parallel or perpendicular coupling). When these effects are chosen to be conflicting (such as commonly used in liquid crystal displays), a transition from low to moderate field can results in the transition of the orientation of the nematic director from the anchored state to the field aligned state (Fréedericksz and Repiewa 1927). However, applying much higher electric fields leads, for certain field coupling parameters, to electrohydrodynamic (EHD) instabilities, which are electric-field-induced phenomena that are caused by the flow of the liquid crystal (Blinov 1986, 1998). Due to the flow distortion of the director alignment, the instability is usually involving characteristic optical patterns, while the symmetry of the cuvette geometry, the symmetry-breaking elements, and the optical properties of the sample can all result in very different patterns and corresponding threshold values (Dubois-Violette et al. 1971; Kai et al. 1996). Similar electroconvective instabilities were reported with the field being applied transversally to the confining plates, however this situation is in comparison poorly documented and usually ignored in theoretical works and reviews (Williams 1972; Raghunathan et al. 1992).

In this work, we report the observation of electrohydrodynamic instabilities in an initially cholesteric suspension of CNCs in toluene when submitted to high AC electric fields applied in the transverse direction, namely parallel to the confining transparent walls of a glass cuvette. This article is organized as following. First, the materials and methods are described, including the different observation setups. Second, a qualitative description of the instabilities is presented, combining direct observations and light scattering patterns. Finally, a brief discussion of these observations is then proposed, along with tentative explanations. In the conclusions, we propose some directions for future experimental investigations that should help clarifying the underlying mechanism of this instability.

## Experimental methods

### Preparation of CNC suspension in water

An aqueous CNC suspension was obtained from the acid hydrolysis of cotton linters in 64 wt% sulfuric acid for 30 min at *T* = 63 °C, following the method initially described by Revol et al. (1994) The suspensions were washed by repeated centrifugation/redispersion steps, dialyzed against distilled water until constant conductivity of the dialysis bath and ultrasonicated for 4 min (Branson Digital Sonifier 450). After filtration (0.8 μm, cellulose nitrate membranes, Sartorius), stable aqueous suspensions of rodlike CNCs were obtained. Conductometric titration against NaOH was used to quantify the functionalization with −OSO_3_^–^ groups of the resulting CNCs, leading to 181 mmol S kg^−1^ (i.e. [S] ≈ 0.58 wt%). This same batch was used in this previous publication from the same authors (Frka-Petesic et al. 2017).

### Preparation of CNC suspensions in toluene

CNC suspensions in toluene were obtained by adding Beycostat NA surfactant (BNA, CECCA-ATO, Co.), a phosphoric ester of polyoxyethylene (9) nonylphenyl ether, with a surfactant/CNC ratio of 4/1 (w/w) to the aqueous CNC dispersion. After freeze-drying, the solid residue was redispersed in toluene with sonication. To remove the excess surfactant, the suspension was centrifuged, and the resulting pellet was collected and redispersed in toluene, centrifuged once more, and this second pellet was considered as the starting paste to prepare other samples. To produce a 40 wt% concentrated homogeneous stock suspension, some paste was redispersed in toluene using a planetary centrifugal mixer (Thinky Mixer ARE-250, Poly Dispensing System). Investigated samples resulted from the dilution of such stock suspension. The sample used in this work was also used in a previous work, where it was labeled “sample A-1” (Frka-Petesic et al. 2017).

### Preparation of electric field cuvettes

Large, rectangularly profiled borosilicate glass capillaries (CM Scientific Rect. Boro Capillaries, #4410-100, length 100 mm, internal width 10.0 mm, external width 11.4 mm, internal thickness 1.0 mm) were silanized (i.e., covalently grafted with hydrophobic organosilane groups) using a standard procedure [for example, see (Szkop et al. 2018)] and left to dry in the oven. Note, this procedure was not essential here and was only made to avoid adhesion of the sample to the capillary walls in the case of samples meant to be photopolymerized through solvent crosslinking (which is not the case of CNCs in toluene). The capillaries were split into two halves under an acetylene flame, and a pair of stainless-steel wires (diameter 0.9 mm) were inserted inside each half-capillary in order to be used as electrodes (each half-capillary making an electric field cuvette). Electric terminal blocks connectors were mounted on the wire ends to allow easy and secure connection with the high voltage source.

### Sample preparation in electric field cuvettes

Apolar CNC suspensions were inserted into the electric field cuvette with a syringe and a needle and sealed with a Teflon-based vacuum grease (Fomblin PFPE—Solvay) to prevent evaporation and leakage. The filling level of the cuvette was marked to monitor any sign of solvent evaporation and thus concentration shift. The dry residue of the sample was used to determine dry mass by weighing. The CNC concentration of the sample was then corrected from the dry mass estimation and any reduction in sample volume. The samples were left at rest in these cuvettes for at least 48 h prior to any experiment to allow for possible phase separation to occur.

### Generation of strong electric fields

The electric field was generated by a function generator as a 1 kHz sinusoidal AC signal at low voltage, and then amplified with a Trek model 10/10B high voltage power amplifier. The voltage was controlled via a bespoke program controlling the function generator (using Delphi, via an RS232 port). The reported AC electric field value corresponds to the rms field given by $E = {V}_{\mathrm{rms}}/d$, where $d$ is the gap between the electrodes and ${V}_{\mathrm{rms}}={V}_{\mathrm{pp}}/2\sqrt{2}$, with ${V}_{\mathrm{pp}}$ the peak-to-peak voltage value displayed by the function generator.

### Direct sample observation under electric fields

**Fig. 1:**
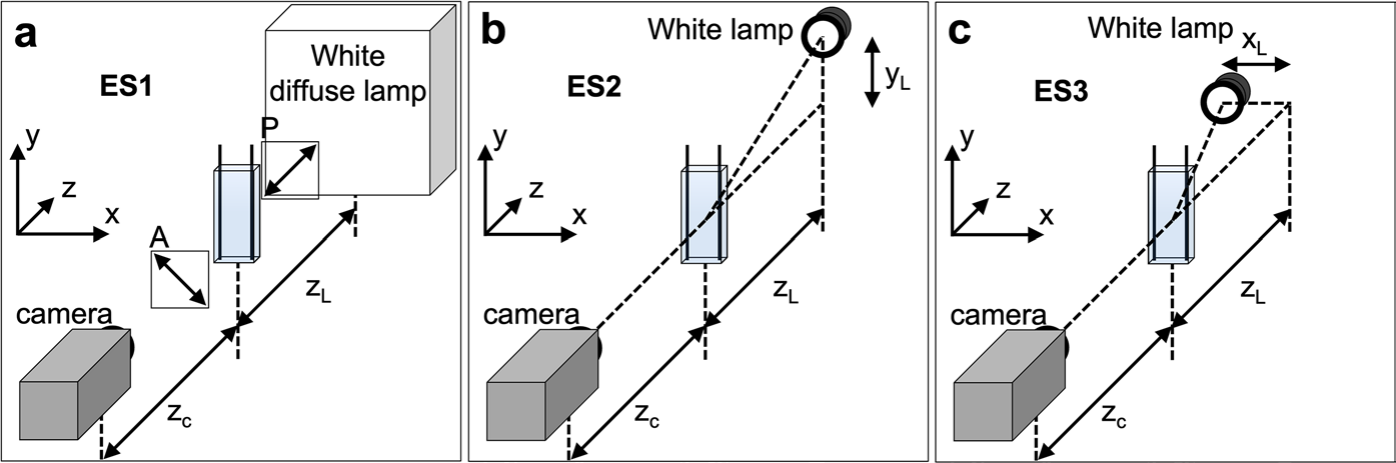
Schematics describing the experimental setups used for direct observation.** a** ES1, for observation between crossed polarizers.** b** ES2, for asymmetrical oblique illumination in transmission, with the light source in the ($\mathbf{y},\mathbf{z}$) plane and** c** ES3, with the light source in the ($\mathbf{x},\mathbf{z}$) plane. The AC electric field is applied in the x direction (between the two vertical electrodes inside the cuvette, in black)

The sample was monitored under an electric field using several complementary methods, including observation in various illumination conditions, listed as experimental setups ES1 to ES1 and described in Fig. 1. Photographs or videos were acquired using either a color camera (Sony XCD- SX90CR, with SonyZCL Viewer software, for photos only) or a monochrome camera (Chameleon, FlyCap software, for photos and videos, settings: exposure value EV = 1.91, auto-unchecked, planar image format 640 × 480 Y8 (8 bits), shutter 110 ms, 1.875 fps (slow) or 7.5 fps (fast), no gain. fps = frame per seconds). Images were processed with the ImageJ software.

### Indirect sample monitoring by laser diffraction under electric fields

**Fig. 2:**
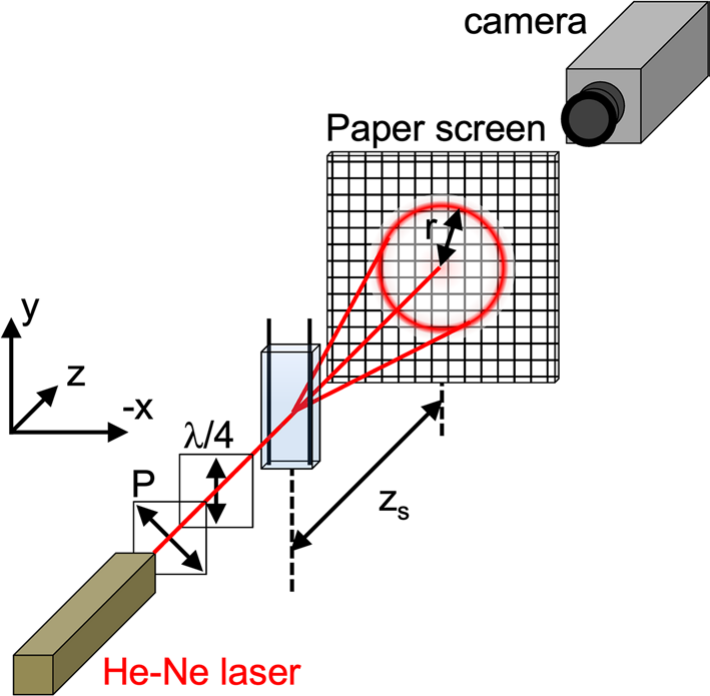
Schematics describing the experimental setup ES4 for the observation of the laser diffraction pattern under electric fields. The distance ${\mathrm{z}}_{\mathrm{s}}$ was varied to access different ranges of scattering vector on the screen

The sample was also investigated using laser diffraction using the experimental setup ES4, as described in Fig. 2. In this configuration, the laser beam of a He–Ne linearly polarized laser was first passing through another polarizer P, and then through a quarter-waveplate (λ/4) with a slow axis aligned at 45° to the axis of P to produce circularly polarized light (however, the handedness was not determined). The diffraction patterns were acquired using a monochrome camera (Chameleon, see details above) placed in the back of a paper screen.

The use of circularly polarized light might be surprising and requires some explanations. It was used to suppress the linear polarization of the laser, which causes a problem for further data interpretation, especially for the study of the unwinding of the cholesteric structure into a nematic order at moderate electric field intensity. Indeed, since cholesteric suspensions with a pitch in the micron range diffract light mostly at grazing angles in transmission, this grazing condition mainly diffracts the linearly polarized light perpendicular to the helical axis direction. The azimuthal variation of the diffracted light intensity would then be biased by the linear polarization of the laser source instead of solely due to a different angular distribution of such diffracting domains. In the reported dataset, the handedness of the circular polarization used in the setup was not determined. However, this is expected to have no direct implication since the left-handed cholesteric suspension is completely unwound into a nematic-like order above 0.4 kV/cm rms.

## Results

### Sample properties

The behavior of the CNC suspension at low and moderate electric fields (up to 0.6 kV/cm) was explored experimentally and described in a previous publication (Frka-Petesic et al. 2017). The studied sample was prepared by dilution of the initial concentrated paste and then allowed to rest for two days in a vertical position (see sample properties summarized in Table 1). This allowed for the phase separation of the CNCs into a biphasic sample, with the denser cholesteric phase accumulating at the bottom and the isotropic phase on the top. In the following, the observation of the sample is reported using direct observation between crossed polarizers, direct observation in oblique illumination in transmission, and laser diffraction.

**Table 1:** Sample properties and characteristic observations of its behavior under increasing electric field

c (wt%)	% aniso	Pitch (µm)	${E}_{un}$(kV/cm)	${E}_{in}$(kV/cm)
28.8%	70%	4.57	0.394	4.6 ± 0.2

Note, the mass fraction $\mathrm{c}$ (wt.%) includes the surfactant coating onto the CNCs. The pitch refers to its pitch in the absence of field, the field ${\mathrm{E}}_{\mathrm{un}}$ denotes the field above which the cholesteric fully unwinds and ${\mathrm{E}}_{\mathrm{in}}$ the field above which the instability is observed at 1 kHz

### Observation between crossed polarizers

**Fig. 3:**
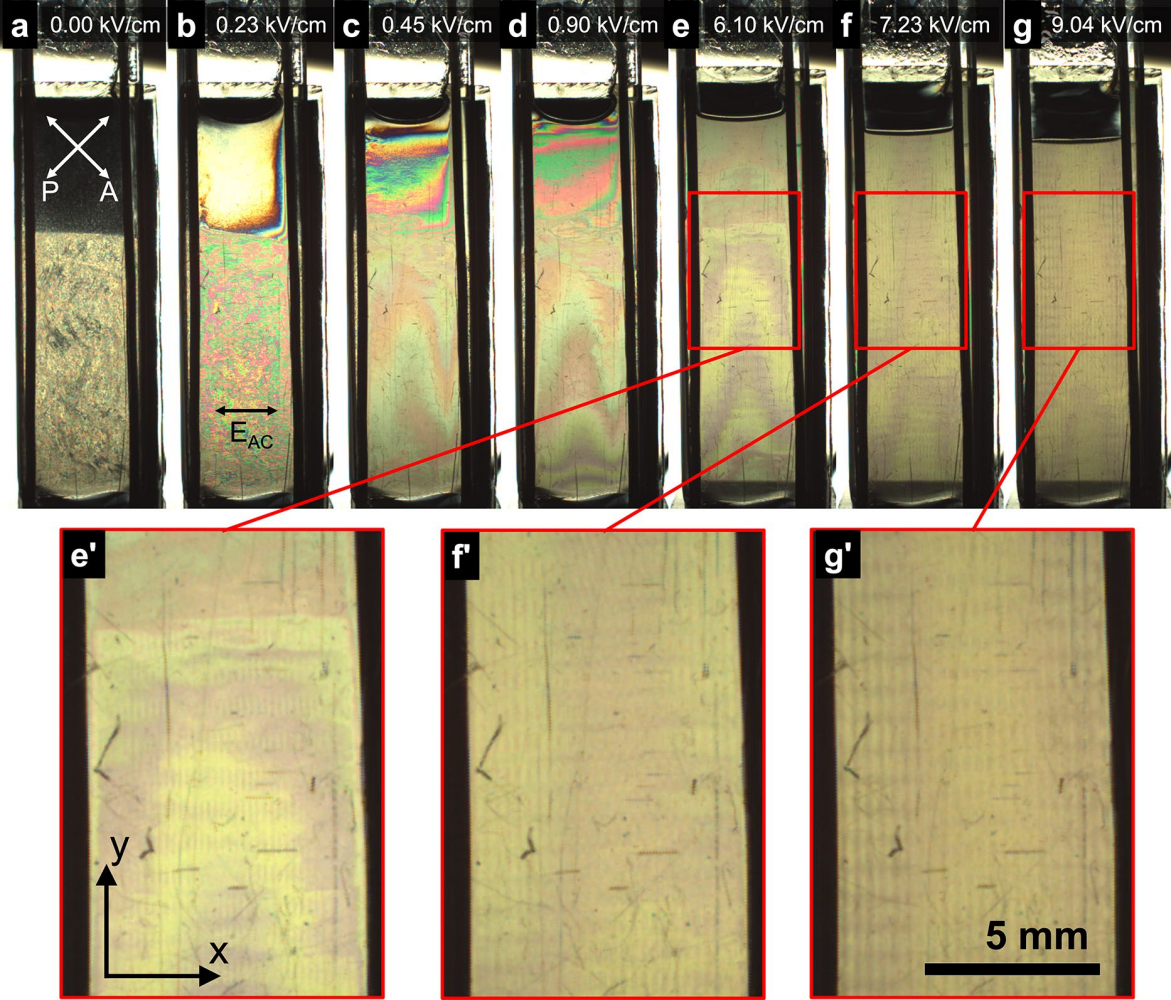
Observation of the sample between crossed polarizers, using the experimental setup ES1. **a** Observation of the sample left overnight at rest (the sample was exposed to various electric field sequences the day before). **b**–**g** Observations under increasing electric fields. These images were recorded within a window of 4 h from the beginning of the experiment. **e'**–**g’** insets with magnified views. The electrodes are visible as the two black vertical wires on each side of the cuvette

These two phases are easily distinguished when the sample is imaged between crossed polarizers, as the isotropic phase is not birefringent and appears dark, while the cholesteric phase is birefringent, yet with a disordered polydomain structure. A photograph of the sample taken after exposure to various electric fields and then an overnight rest in the absence of a field is shown in Fig. 3a. In this geometry, the electrodes are placed vertically and parallel to one another, so that the field is applied in the $\mathbf{x}$ direction, while the sample is imaged in the ($\mathbf{x},\mathbf{y}$) plane. The sample was first observed under electric field using the experimental setup ES1 described in Fig. 1a, where the sample was placed between crossed polarizers in front of a white diffusing light source. The application of an electric field led to several visual effects, as shown in Fig. 3, that depend both on the strength of the field and, especially for weaker fields, on the history of the electric field sequence applied to the sample. Initially, the application of a field of 0.23 kV/cm at 1 kHz caused the isotropic phase to become bright. At 0.45 kV/cm, Newton colors were already well developed, typical of the Michel–Lévy chart, with a gradient of its corresponding birefringence, indicative of a gradient of CNC concentration towards the bottom phase. In the bottom phase, the granular texture evolved from 0 to 0.23 kV/cm into a polydomain structure of growing domains with the appearance of Newton colors. This is consistent with the alignment of CNC at a higher concentration by the reorientation of the cholesteric domains by the electric field as described in the previous work, with their helical axes perpendicular to the electric field (Frka-Petesic et al. 2017). The interface between the two phases gradually disappeared as the field intensity increased and eventually vanished completely.

Importantly, the sample develops regular bands, visible in Fig. 3e–g and corresponding magnified views, with a periodicity in the $\mathbf{x}$ direction but also in the **y** direction. Note that the liquid–air interface between the two electrodes initially showed a curved meniscus that became more and more straight as the field increased. Note, the vertical position of this meniscus also moved downward, as part of the suspension was displaced away from the electrode gap towards the two small outer interstices behind each electrode and the cuvette walls. After the field was reduced to lower field values, the sample appeared homogeneous and did not visibly macro-phase-separate within ca. 10 min (delay before the next experiment).

### Observation in asymmetrical oblique illumination in transmission

**Fig. 4:**
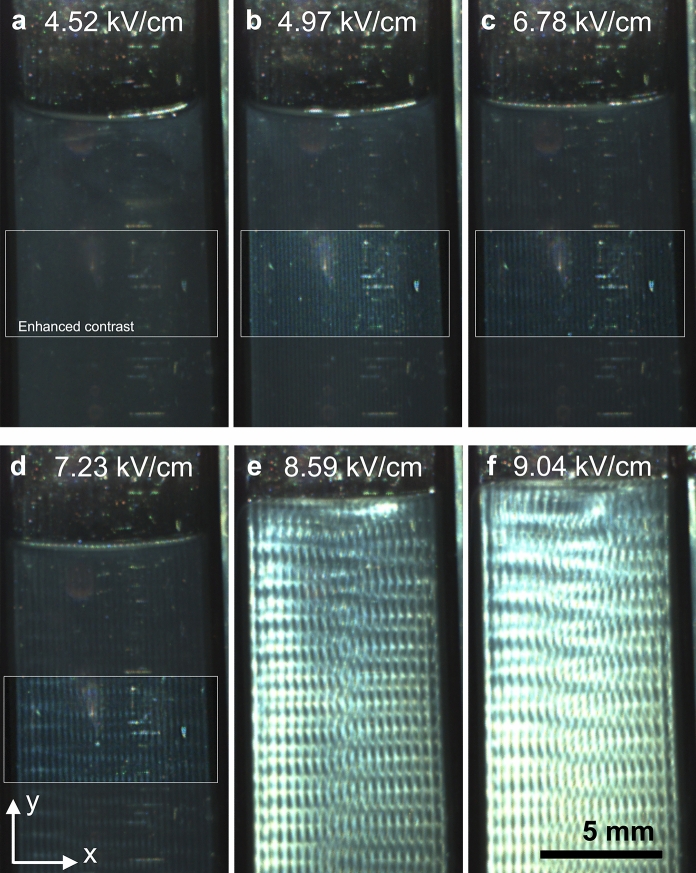
Observation of the sample under asymmetrical oblique illumination in transmission, using the experimental setup ES2, so the illumination is in the ($\mathbf{y},\mathbf{z}$) plane. **a** No visible pattern. **b** A simple pattern of vertical periodic stripes appears, made more visible in the box with enhanced contrast. **c**–**d** A second modulation in y appears gradually. **e**–**f** The pattern evolves towards doubling the periodicity in y and becomes much brighter, with some coupling between the x and the y periodicities, with tilted structures

The sample was then imaged using the experimental setup ES2, as described in Fig. 1b. In this configuration, the polarizers were removed, and the sample was observed with a localized light source placed in the background, in the ($\mathbf{x},\mathbf{z}$) plane. The sample appeared uniform up to 4.52 kV/cm, while vertical bands appeared at 4.97 kV/cm, with a periodicity in the $\mathbf{x}$-axis, as shown in Fig. 4. The periodicity of these bands increased with the field amplitude. At 6.78 kV/cm, these bands developed an additional modulation along the $\mathbf{y}$-axis (Fig. 4c). This vertical modulation was also visible in Fig. 3e’–g’ using the previous setup. Interestingly, the modulation in the $\mathbf{x}$ and the $\mathbf{y}$ axes started to couple and developed a crossing pattern with tilted bands (Fig. 4d). The liquid–air interface also showed periodic bright and dark spots, with the same periodicity as the period in the $\mathbf{x}$-axis, suggesting a sinusoidal modulation of the free interface by the electric field. At 8.59 kV/cm, the pattern became much brighter, and the periodicity along the $\mathbf{y}$-axis seems to have doubled as the horizontal lines apparently grouped in pairs (Fig. 4e–f). While the horizontal lines from each electrode propagated towards the center of the cuvette, they seem to be in antiphase and meet in a central periodic pattern with a regular yet unclear structure. The pattern became much brighter at higher fields, i.e., from 7.5 kV/cm onwards. Interestingly, when the sample was then imaged using the experimental setup ES3, as described in Supplementary Information, Figure S1, where the illumination, characterized by an incident wavevector ${\mathbf{k}}_{\mathbf{i}}$ placed in the ($\mathbf{x},\mathbf{y}$) plane as opposed to the ($\mathbf{x},\mathbf{z}$) plane, the instability was barely visible. Since a nematic order of aligned CNCs scatters light mostly in the plane (${\mathbf{k}}_{\mathbf{i}}$, ${\mathbf{k}}_{\mathbf{i}}\times \mathbf{n}$) perpendicular to the local director $\mathbf{n}$, this plane contains the $\mathbf{z}$-axis in the ES2 but not in the ES3, explaining the difference of contrast between the two datasets. This also indicates that the director $\mathbf{n}$ was modulated in the sample, which we ascribe to some electrohydrodynamic convection instability.

**Fig. 5:**
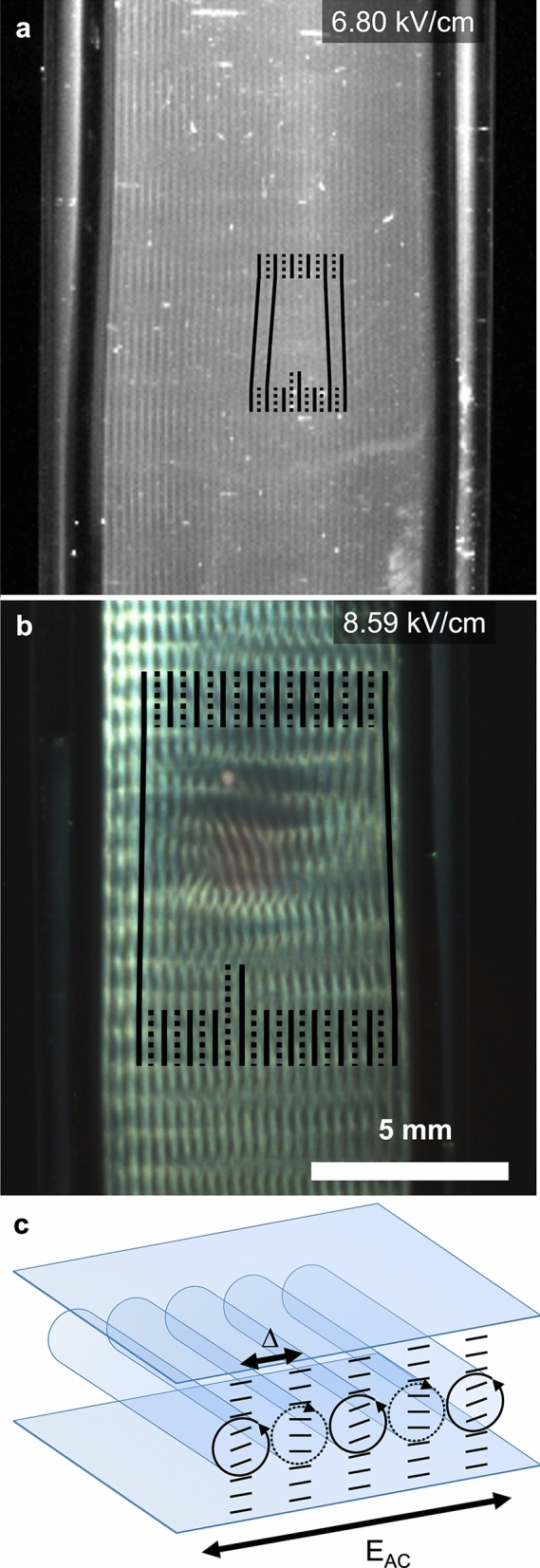
Observation of defects in the periodic patterns, using the experimental setup ES2. The defects always admit a pair of bright lines (two bright and two dark). **a** Snapshot from video 1 (Online Resource 2, monochrome camera, 1.875 fps, frame #1022) of a defect in the normal roll geometry. **b** Photograph of a defect where the pattern couples between x and y periodicities. The full and dotted lines are guide to the eye to distinguish the insertion of a pair of vertical bright lines. **c** Schematic of the contra-rotative “normal rolls” and their proposed influence on the director field, shown as short black lines

The periodicity of the pattern appears to be selected as a submultiple of the gap between the electrodes. As the electric field varies, the periodicity of the pattern changes. Since the wire electrodes were not perfectly straight, the gap between the electrodes was not everywhere the same. This also led to local variations of field intensity, since the field is defined as the voltage divided by the electrode gap. These two effects caused the periodicity of the pattern between the electrodes to vary by the appearance and the continuous displacement of a disclination in the pattern from one end of the electrode pair to the other one, propagating vertically, resulting in the insertion or the removal of a pair of dark and bright lines (i.e., two dark and two bright lines) between the electrodes. Such disclination lines are illustrated in Fig. 5a–b and in video 1 (Online Resource 2), where the field is varied from 0.907 kV/cm to 9.07 kV/cm at a constant pace). The systematic insertion of additional lines in pairs (unless these insertions occur from one electrode) is consistent with ascribing these bands as contra-rotative rolls, common in electrohydrodynamic convection instabilities and often referred to as “Williams striation” or “normal rolls” and schematically illustrated in Fig. 5c (Williams 1963; Goossens 1978). The periodicity of the structure is then twice the periodicity $\Delta$ of the pattern. Interestingly, the development of a more complex pattern, as visible in Fig. 5, comes with a distortion of the pattern on a larger area, scaling from only 1 mm for normal rolls (Fig. 5a) to about 3–5 mm in the case of a 2D grid-like pattern (Fig. 5b).

### Observation by laser diffraction

**Fig. 6:**
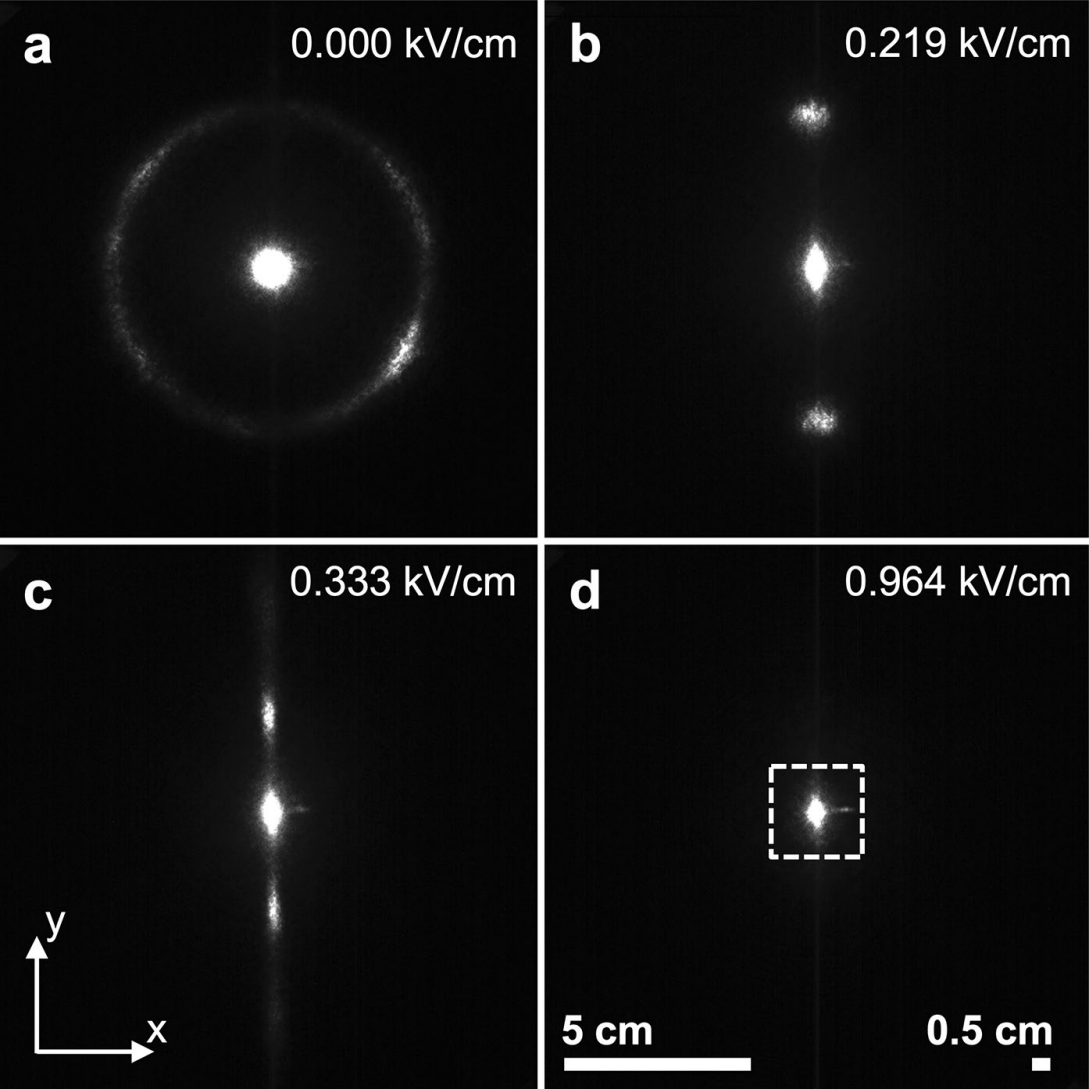
Laser diffraction pattern taken using the experimental setup ES4 (the AC electric field is applied in the $\mathbf{x}$ direction), showing the alignment of the CNC suspension at low to moderate electric fields, before the instability is observed. **a** Initial diffraction pattern from the polydomain cholesteric structure at rest, **b** pattern indicating the reorientation of the helicoidal domains, **c** distortion and unwinding of the cholesteric structure, **d** completely unwound structure into a nematic order. The distance from sample to screen was ${\mathrm{z}}_{\mathrm{s}}$ = 15 cm and the scale bar indicates the distances on screen (e.g., 5 cm corresponds to $\mathrm{q}$ $\approx$ 3.18 µm^−1^, or $2\uppi /\mathrm{q}$ $\approx$ 1.97 µm). The white dashed square highlights the range of scattering wavevector investigated in Fig. 7

The sample was then investigated using laser diffraction using the experimental setup ES4, as described in Fig. 2. In the absence of an electric field, the diffraction pattern of the suspensions forms a diffraction ring. The radius of the ring, observed on a screen placed at a distance ${z}_{s}$ from the sample, corresponds to a diffraction angle ${\theta }_{\mathrm{exp}}=\mathrm{atan}\left(r/{z}_{s}\right)$, and this angle is related, after Snell’s law correction at the sample-air interface ($n \mathrm{sin}\theta =\mathrm{sin}{\theta }_{\mathrm{exp}}$, with $n\approx 1.5$ the optical index of the sample), to the scattering wavevector $q=\left(4\pi n/\lambda \right)\mathrm{sin}\left(\theta /2\right)$. The azimuthal profile of the diffracted intensity, whose variation can be expressed from the cartesian coordinates ($x,y$) to the polar coordinates $r=\sqrt{{x}^{2}+{y}^{2}}$ and $\varphi =\mathrm{atan}\left(y/x\right)$, is initially constant and results in a diffraction ring, in agreement with a cholesteric polydomain structure with no preferential direction of alignment. As described in our previous work, the application of an increasing AC electric field leads initially to a reorientation of the domains, visible as the azimuthal profile evolves to a pair of diffraction peaks. Upon further increase in the electric field intensity, a distortion and unwinding of the cholesteric into a nematic-like order is observed, which is indicated by a decrease of the peak diffraction angle (showing a diverging pitch). Finally, complete unwinding is shown by the disappearance of the diffraction peaks above a critical electric field ${E}_{un}$ (*un* standing for ‘unwound’, see Table 1 for values, experimental data available in our previous work) (Frka-Petesic et al. 2017). As shown in Fig. 6, the diffraction pattern shows no more diffracting structures at $E$ ~ 1 kV/cm, well above ${E}_{un}$.

**Fig. 7:**
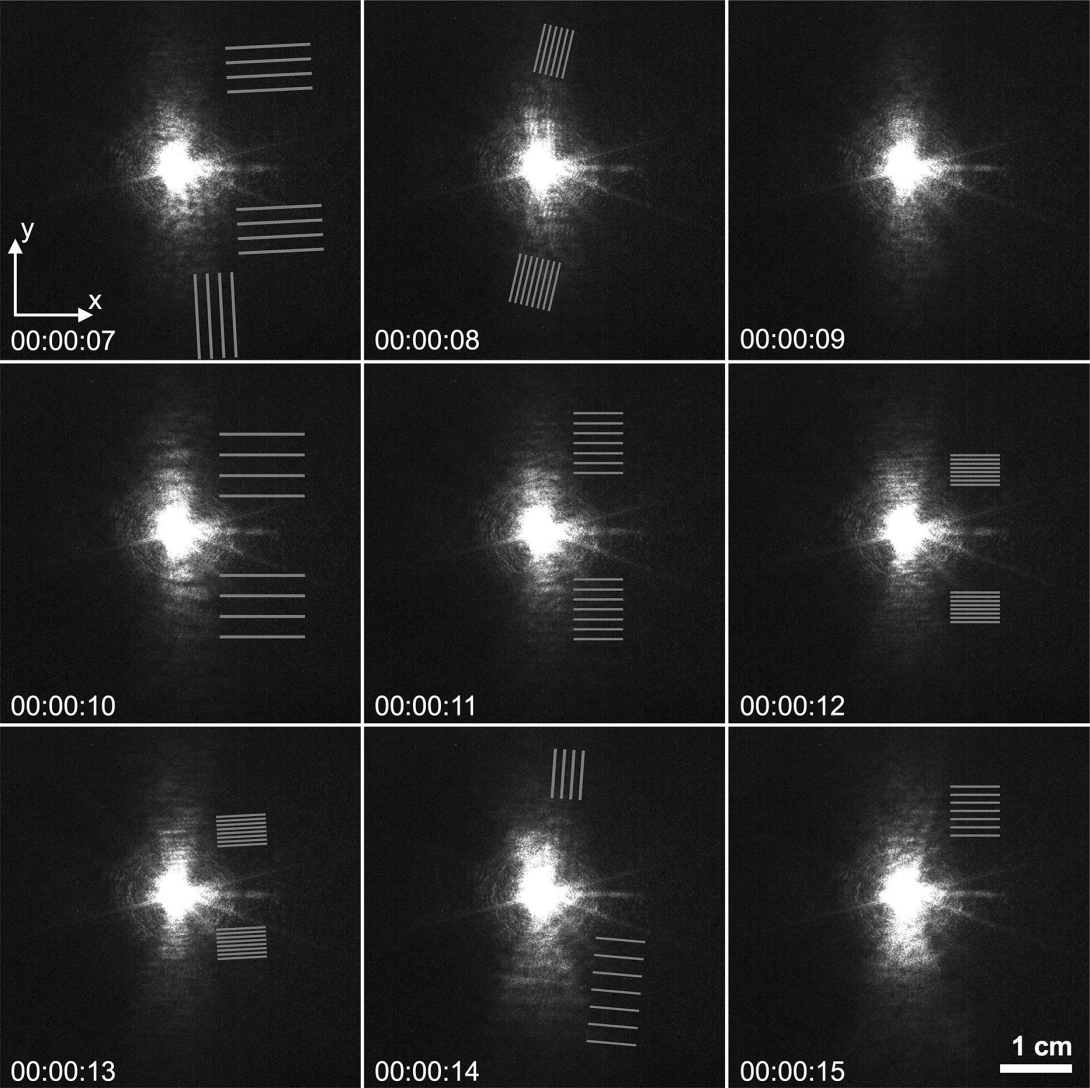
Snapshots from video 2 (Online Resource 3), showing laser diffraction patterns taken using the experimental setup ES4, as the field is varied from 4.3. kV/cm to 6.07 kV/cm. The distance from sample to screen was ${\mathrm{z}}_{\mathrm{s}}$ = 31 cm and the scale bar indicates the distances on the screen (e.g., 0.1 cm corresponds to $\mathrm{q}$ $\approx$ 0.032 µm^−1^, or $2\uppi /\mathrm{q}$ $\approx$ 195 µm). The grey stripes are guide to the eye to distinguish the periodicities of the stripes

The laser diffraction pattern reveals some surprising patterns at very low angles when the AC electric field is applied at much higher values, i.e., at values where the patterns were previously observed using the experimental setup ES2. As reported in Fig. 7, the diffraction pattern presents some transient periodic bands of various orientations and periodicities, mostly visible when the field varies from one to another high value.

The video 2 (Online Resource 3), from which the snapshots in Fig. 7 were extracted, reveals more clearly the transient nature of these bands. More than the extracted snapshots, the video gives the impression of Moiré patterns observed when different periodic patterns are overlayed. From the calibration of the k-space of the diffraction pattern, the associated periodicities were estimated to range from $2\pi /q$ = 70–540 µm. We suspect that these transient patterns are related to the brief passing of the defects reported in Fig. 5a–b through the illuminated area of the laser as the field is altered. Indeed, the video 1 (Online Resource 2) shows defects traveling vertically as the field is varied, which means that at a certain point in time these defects are expected to move across the sample region illuminated by the laser beam while the diffraction pattern is being recorded.

## Discussion

The observations of the patterns in the high field regime strongly suggest some electrohydrodynamic (EHD) instabilities, also sometimes referred to as electroconvection instabilities. In the range of electric fields applied here, the cholesteric organization is completely unwound, and although the chiral nature of the system remains, it is likely that it does not play an important role in the observed phenomena, at least for the onset of the instability of the so-called *normal roll* pattern. The normal roll pattern is made of pairs of contra-rotating rolls, which is in agreement with the insertion of a pair of dark and bright lines (i.e. two dark and two bright lines) when a defect is introduced, as shown in Fig. 5.

To our knowledge, the electrohydrodynamic convection instabilities reported in this work are the first example of such behavior produced with colloidal suspensions of cellulose nanocrystals. However, these instabilities have been broadly studied in the past century on molecular liquid crystals in a very different geometry, whereby the field is applied parallel in the direction of highest confinement, parallel to the viewing direction by using transparent electrodes, and the variety of patterns observed in this configuration already justified an effort of classification (Blinov et al. 1982; Blinov 1986, 1998). The physical interpretation of the underlying mechanisms for this instability is usually captured by the now standard Carr-Helfrich mechanism (Carr 1963; Helfrich 1969), which was successfully described theoretically (Dubois-Violette et al. 1971; Smith et al. 1975) and confirmed by experiments (Orsay Liquid Crystal Group 1970). It is usually divided in a conductive regime at lower frequencies and a dielectric regime at higher frequencies. In the conduction regime, electric charges oscillate while the director distortion remains essentially stationary. In the dielectric regime, the director oscillates while the charges remain nearly stationary. Instead, the literature reporting electroconvection instabilities observed by applying a field in the in-plane direction (in the $\mathbf{x}$-axis as opposed to the $\mathbf{z}$-axis), is surprisingly rare, which complicates the search for relevant theoretical or experimental analogs of the observations reported in this work. As such, the example of an in-plane field applied to a nematic structure with a negative dielectric anisotropy (i.e. with the director $\mathbf{n}$ aligning away from the field $\mathbf{E}$) was reported to form “normal rolls” aligned in the direction *parallel* to the electric field, and *perpendicular* to the director $\mathbf{n}$(Williams 1972; Raghunathan et al. 1992). However, the theoretical treatment proposed for these observations was however still the Carr-Helfrich mechanism, with a twist-bend deformation of the director away from its equilibrium position, and a similar range of instability for the electric field and frequency applied (Raghunathan et al. 1992).

In the case of the present work, however, the dielectric anisotropy is positive (i.e. with the director $\mathbf{n}$ aligning in the field $\mathbf{E}$, and $\mathbf{E}$ is applied longitudinally along $\mathbf{x}$. This implies that the director $\mathbf{n}$ before the onset of the instability is *parallel* to $\mathbf{E}$, unlike in the Carr-Helfrich mechanisms, making these cases not directly applicable to the analysis reported in this work.

Beyond the standard Carr-Helfrich instability, non-standard mechanisms involving additional effects such as flexoelectricity, have also been reported (Buka et al. 2007). The flexoelectric effect occurs when the particles composing the liquid crystal bare an electric dipole (or a quadrupole), which results in the appearance of localized charges from the distortion of the director (Meyer 1969; Chandrasekhar 1992). The CNCs display a permanent dipole behavior under field reversal, suggesting this effect may play a dominating role. A more quantitative analysis of the instability reported in this work would allow us to explore these aspects and would be suitable for future work.

## Conclusions

In this work, spontaneous periodic patterns were observed in apolar CNC suspensions exposed to strong AC electric field. Different qualitative observations strongly suggest that the origin of these patterns is an electrohydrodynamic (EHD) convection instability, also sometimes referred to as an electroconvection instability. Most common geometries for such observations are different from the geometry used here, which does not allow for a simple interpretation based on the existing literature. While the exact mechanism of this instability remains unclear, it is believed to produce a pattern of pairs of contra-rotative convection rolls at the onset of the instability. A quantitative characterization would allow for a better understanding of the origin of this instability, but also help identifying the key properties that make cellulose nanocrystals such a suitable system for exploring these collective behaviors.

## Supplementary Information

Below is the link to the electronic supplementary material.

Supplementary file 1 (PDF 2908 KB)

Supplementary file 2 (AVI 450390 KB)

Supplementary file 3 (AVI 720017 KB)

## Data Availability

Supplementary Information is available online, containing a pdf document (Online Resource 1), the video 1 (Online Resource 2) and the video 2 (Online Resource 3). Additional data related to this publication are available free of charge at the University of Cambridge data repository (10.17863/CAM.99836) or from the authors.
