# Supplementary material for: Electrohydrodynamic convection instabilities observed in suspensions of cellulose nanocrystals
Source: Cellulose (Lond). 2023 Jul 26;30(13):8311–23. doi: 10.1007/s10570-023-05391-6 (PMC10473993; doi:10.1007/s10570-023-05391-6)
Supplement: Supplementary file 1 — (PDF 2908 KB) [file 10570_2023_5391_MOESM1_ESM.pdf]

Supplementary Information

for

Electrohydrodynamic Convection Instabilities Observed in Suspensions of Cellulose Nanocrystals

Bruno Frka-Petesic,* Bruno Jean, Laurent Heux*

Cellulose, DOI: 10.1007/s10570-023-05391-6

Dr. Bruno Frka-Petesic

Yusuf Hamied Department of Chemistry, University of Cambridge, Lensfield Road, CB2 1EW, United Kingdom

International Institute for Sustainability with Knotted Chiral Meta Matter (WPI-SKCM^2), Hiroshima University WPI, 1-3-1 Kagamiyama, Higashi-Hiroshima City, Hiroshima 739-8526, Japan

Dr. Bruno Jean, Dr. Laurent Heux

Univ. Grenoble Alpes, CNRS, CERMAV, 38000 Grenoble, France

e-mails: BFP: bf284@cam.ac.uk, LH: heux@cermav.cnrs.fr

Keywords: Cellulose nanocrystal, colloidal liquid crystal, cholesteric, electroconvection.

Note, additional data related to this publication are available free of charge at the University of Cambridge data repository (http://dx.doi.org/10.17863/CAM.99836) or from the authors.

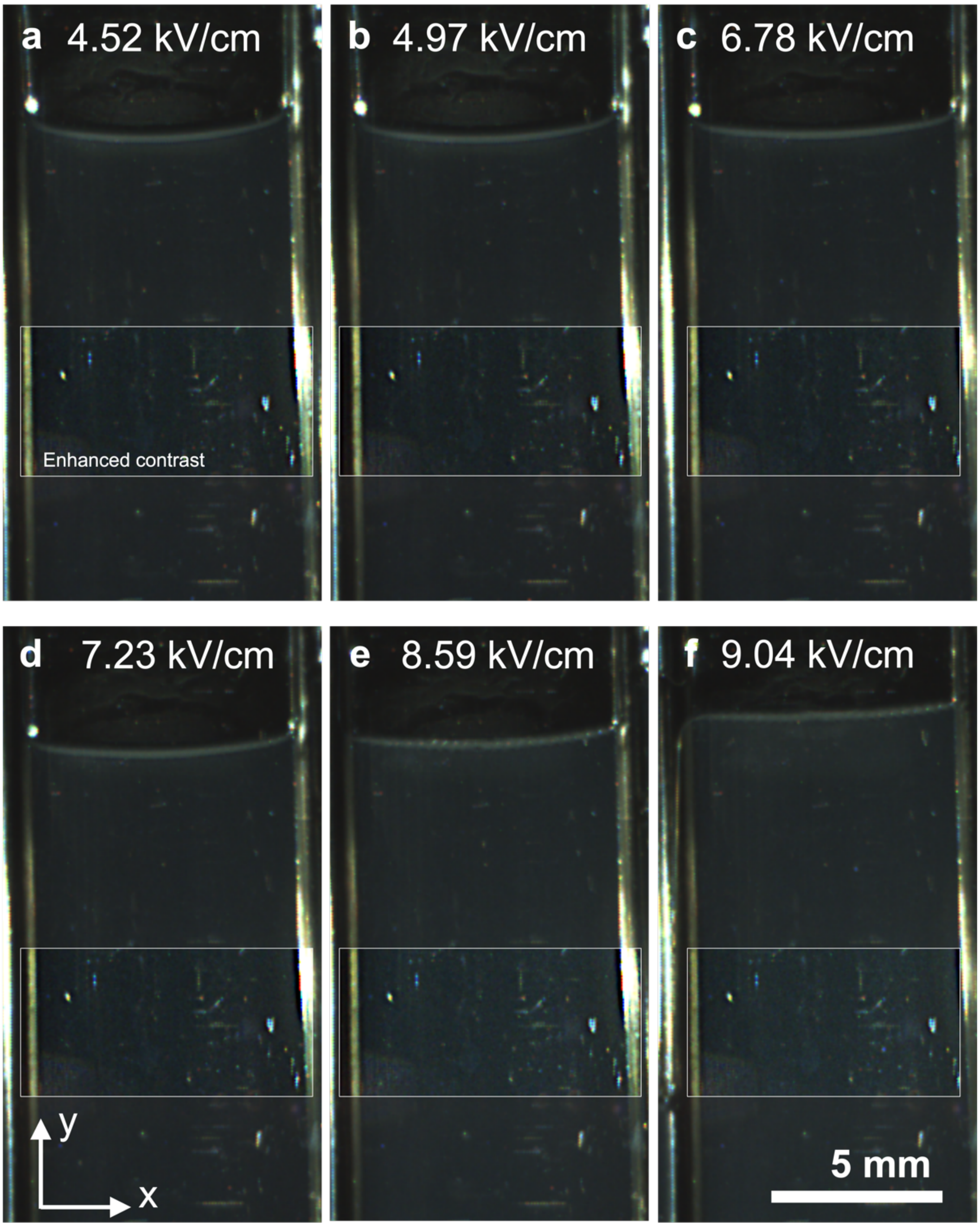

Figure S1. Observation under asymmetrical oblique illumination in transmission, taken at the same field values as in Figure 4 but using the experimental setup ES3 instead of ES2, so that the illumination is in the (**x**, **z**) plane. **a.** no visible pattern, as expected from Figure 4a. **b-f.** no pattern is easily distinguishable, despite being seen at the same conditions using ES2. The distortion of the meniscus from curved to almost straight clearly shows that a strong electric is applied. The periodic bands are barely noticeable in figure (e), and by the periodic fringes of the meniscus.
